# Antihypertensive drugs are associated with reduced fatal outcomes and improved clinical characteristics in elderly COVID-19 patients

**DOI:** 10.1038/s41421-020-00221-6

**Published:** 2020-10-29

**Authors:** Feifei Yan, Fengming Huang, Jun Xu, Penghui Yang, Yuhao Qin, Jingjun Lv, Shaogeng Zhang, Lu Ye, Ming Gong, Zhibo Liu, Jie Wei, Tuxiu Xie, Kai-Feng Xu, George F. Gao, Fu-Sheng Wang, Lin Cai, Chengyu Jiang

**Affiliations:** 1 Department of Orthopedics, Zhongnan Hospital of Wuhan University, Wuhan, Hubei 430071 China; 2 Department of Medical Quality Control, Leishenshan Hospital, Wuhan, Hubei 430000 China; 3 The State Key Laboratory of Medical Molecular Biology, Institute of Basic Medical Sciences, Chinese Academy of Medical Sciences, Department of Biochemistry, Peking Union Medical College, Beijing, 100005 China; 4 Emergency Department, Peking Union Medical College Hospital, Chinese Academy of Medical Sciences, Beijing, 100730 China; 5 Fifth Medical Center of Chinese PLA General Hospital, Beijing, 100039 China; 6 Emergency Department, Renmin Hospital of Wuhan University, Wuhan, Hubei 430060 China; 7 General Medicine Department, Guang-gu Division of Renmin Hospital of Wuhan University, Wuhan, Hubei 430200 China; 8 Department of Pulmonary and Critical Care Medicine, Peking Union Medical College Hospital, Chinese Academy of Medical Sciences, Beijing, 100730 China; 9 CAS Key Laboratory of Pathogenic Microbiology and Immunology, Institute of Microbiology, Chinese Academy of Sciences, Beijing, 100101 China

**Keywords:** Autoimmunity, RNAi

## Abstract

The novel coronavirus (CoV) severe acute respiratory syndrome (SARS)-CoV-2 outbreak began at the end of 2019 in Wuhan, China, and has spread to over 200 countries. In this multicenter retrospective study, we identified 2190 adult patients admitted for laboratory-confirmed COVID-19 in three participating centers. Multivariate logistic regression was conducted in patients with comorbid hypertension to examine the potential association between clinical outcomes, disease severity, and clinical characteristics with the use of ACEI, ARB, calcium-channel blockers (CCB), beta-blockers (BB), and thiazide diuretics. The clinical outcome, dyspnea, and fatigue were significantly improved in patients, especially elderly patients who were older than 65 years, who took ARB drugs prior to hospitalization compared to patients who took no drugs. The reduction of disease severity of elderly COVID-19 patients was associated with CCB and ACEI users. Clinical indices, including CRP, lymphocyte count, procalcitonin D dimer, and hemoglobin, were significantly improved in elderly ARB users. In addition, the clinical outcomes were statistically significantly improved in patients who took antihypertension drugs ARB, BB, and CCB after statistical adjustment by all ages, gender, baseline of blood pressures, and coexisting medical conditions. Our data indicate that hypertension drugs ARB, ACEI, CCB, and BB might be beneficial for COVID-19 patients.

## Introduction

At the end of 2019, a cluster of lethal pneumonia cases was reported in Wuhan, China. A SARS-CoV-like coronavirus in the respiratory tracts of patients was soon isolated and the viral genome sequenced; it was later named SARS-CoV-2^1–6^. In severe cases of COVID-19, patients develop acute respiratory distress syndrome (ARDS) and often die with multiorgan dysfunction syndrome (MODS). By March 11, 2020, the virus infection had spread to over 100 countries, and SARS-CoV-2 infection was declared a global pandemic by the World Health Organization (https://www.who.int/news-room/detail/30-01-2020-statement-on-the-second-meeting-of-the-international-health-regulations-(2005)-emergency-committee-regarding-the-outbreak-of-novel-coronavirus-(2019-ncov)).

There has been growing concern about the use of antihypertensives in patients with COVID-19, mostly due to the fact that angiotensin-converting enzyme 2 (ACE2)^1,3,4,7–10^, a negative regulator of the renin–angiotensin–aldosterone system (RAAS), is a co-receptor for viral entry into human cells by SARS-CoV-2. ACE cleaves angiotensin I to generate angiotensin II, whereas ACE2 converts angiotensin II into angiotensin (1–7)^11–13^. By counteracting the action of ACE, ACE2 plays a crucial role in maintaining blood-pressure homeostasis, fluid, and salt balance^14,15^. ACE inhibitor (ACEI) and angiotensin receptor blockers (ARB) are the most commonly prescribed antihypertensive medications^16–19^. A viewpoint “COVID-19 and angiotensin-converting enzyme inhibitors and angiotensin receptor blockers, what is the evidence?” published on March 24, 2020 in *JAMA* pointed out that no clinical data are available^20^. In a special report “renin-angiotensin-aldosterone system (RAAS) inhibitors in patients with COVID-19” published on March 30, 2020 in the *New England Journal of Medicine*, the authors discussed the potential for benefit rather than harm of RAAS blockers in COVID-19, but also pointed out that insufficient data are available to determine whether these observations readily translate to humans, and no studies have evaluated the effects of RAAS inhibitors in COVID-19^21^.

A previous study from this research group investigated the role of RAAS in the acute lung failure caused by SARS-CoV-2 infection^22^. SARS-CoV-2 infection and the spike protein of SARS-CoV-2 reduce ACE2 expression, increase angiotensin II-level signaling through the angiotensin II type 1a receptor (AT1a), promote disease pathogenesis, induce lung edemas, and impair respiratory function^9,12^. We demonstrated that the ARB losartan could attenuate acute lung failure in a mouse model whose condition had been worsened after injection of the SARS-CoV-2 spike protein^9^. We also showed imbalanced RAAS in many predisposing conditions for ARDS, including sepsis, acid aspiration, bacteria, SARS-CoV-2, avian influenza (H5N1 and H7N9) infections, as well as nanoparticle aspiration^9,11–13,23^. These studies suggested that blocking the RAAS pathway and reducing angiotensin II levels could ameliorate lung injury^9,11,12,16,23^.

Observational studies have associated the use of ACEIs and ARBs with improved outcomes in patients with pneumonia^24^. Our previous study reported significantly elevated plasma levels of angiotensin II in COVID-19 patients^8^, again indicating RAAS imbalance in COVID-19. More studies of ACEI/ARBs associated with mortality and morbidity of COVID-19 reported different results recently^25–29^. Here, we conducted a retrospective study to examine the potential association between the use of antihypertensives and COVID-19 disease severity.

## Results

### Clinical characteristics of participants

**Fig. 1:**
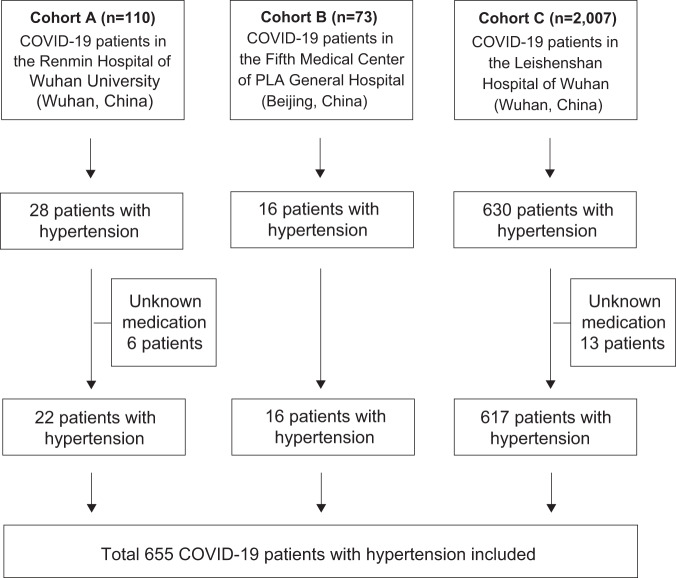
Study design and patient selection. A total of five cohorts (adult patients ≥18 years of age) were included in this retrospective study. Cohort A recorded 28 COVID-19 pneumonia patients with hypertension admitted to the Renmin Hospital of Wuhan University (Wuhan, China) from January 12 to February 9, 2020, cohort B recorded 16 COVID-19 pneumonia patients with hypertension admitted to the Fifth Medical Center of Chinese PLA General Hospital (Beijing, China) from December 27, 2019 to February 29, 2020, and cohort C recorded 630 COVID-19 pneumonia patients with hypertension admitted to the Leishenshan Hospital (Wuhan, China) from February 8 to April 14, 2020. A total of 655 patients with COVID-19 and comorbid hypertension were included in the data analysis.

This study cohort included 2190 patients with COVID-19 who were admitted to three hospitals in China. A total of 655 participants with hypertension were included in the subsequent analysis, and 19 cases were excluded due to missing medication information (Fig. 1). The mean (±SE) age was 64.6 ± 11.8 years, 51.8% of the patients were male (Supplementary Table S1), and 318 patients were >65 years of age (Supplementary Table S2). The distribution of age and sex among total severe (169, 25.8%) and nonsevere (486, 74.2%) COVID-19 patients was significantly different. Severe COVID-19 patients were older (69.6 ± 11.0 years vs 62.9 ± 11.5 years, *P* < 0.001) and more often men (60.4%, 102/169 vs 49%, 238/486, *P* = 0.012). Besides, nonsurvivors were significantly older than survivors (71.0 ± 10.9 years vs 64.2 ± 11.7 years, *P* < 0.001). No association between death and sex was found (*P* > 0.05). Among elderly patients (>65 years), patients who were older were more likely to develop severe COVID-19 and die. Men were more likely to develop severe COVID-19, but this had no statistically significant effect on mortality.

### ARB and CCB are associated with decreased mortality in elderly COVID-19 patients

Using the multivariable logistic regression method, we evaluated the effects of hypertensive comorbidity patients who took antihypertensive drugs prior to hospitalizations, in subgroups, ARB, ACEI, CCB, Thiazide, and BB, on mortality and disease severity compared to a no-drug-used patient group with hypertension comorbidity (Tables 1, 2 and Supplementary Tables S3–S8). The clinical outcomes of ARB users were statistically significantly improved with the adjustment of age, gender, baseline of blood pressure, and coexisting medical condition variables among all patients (adjusted OR = 0.421, 95% CI: 0.19–0.934, *P* = 0.033), as well as in elderly patients who were more than 65 years of age (adjusted OR = 0.202, 95% CI: 0.055–0.745, *P* = 0.016) (Table 1 and Supplementary Tables S5, S6). The clinical outcomes of CCB users were statistically significantly improved in elderly patients (adjusted OR = 0.22, 95% CI: 0.062–0.778, *P* = 0.019) (Table 1 and Supplementary Table S6). Our data indicate that ARB and CCB might be beneficial to COVID-19 patients.

**Table 1:** Association between antihypertensive use and outcome of COVID-19 patients with hypertension comorbidity.

					Unadjusted			Adjusted^a^		
Characteristics		Total patients	Survival	Death	OR	95% CI	*P* value	OR	95% CI	*P* value
All cases, *n* (%)		655	619	36						
	No use	69 (10.5)	62 (10.0)	7 (19.4)	Ref.		Ref.	Ref.		Ref.
	ARB	149 (22.7)	146 (23.6)	3 (8.3)	0.182	0.046-0.727	0.013	0.421	0.19–0.934	0.033
	ACEI	44 (6.7)	43 (6.9)	1 (2.8)	0.206	0.024–1.735	0.147	0.497	0.184–1.34	0.168
	Thiazide	38 (5.8)	33 (5.3)	5 (13.9)	1.342	0.395–4.559	0.751	0.992	0.666–1.477	0.968
	BB	100 (15.3)	97 (15.7)	3 (8.3)	0.274	0.068–1.099	0.093	0.496	0.268–0.919	0.026
	CCB	441 (67.3)	420 (67.9)	21 (5.8)	0.443	0.181–1.085	0.084	0.34	0.119–0.968	0.043
Older than 65 years, *n* (%)		318	292	26						
	No use	31 (9.7)	25 (8.6)	6 (23.1)	Ref.		Ref.	Ref.		Ref.
	ARB	78 (24.5)	76 (26.0)	2 (7.7)	0.11	0.021–0.578	0.006	0.202	0.055–0.745	0.016
	ACEI	19 (6)	19 (6.5)	0 (0)	——	——	0.071	——	——	0.996
	Thiazide	24 (7.5)	22 (7.5)	2 (7.7)	0.379	0.069–2.073	0.443	——	——	0.988
	BB	54 (17)	51 (17.5)	3 (11.5)	0.245	0.057–1.062	0.067	0.531	0.286–0.988	0.046
	CCB	214 (67.3)	198 (67.8)	16 (61.5)	0.337	0.121–0.94	0.043	0.22	0.062–0.778	0.019

^a^Fully adjusted model includes the following covariates: age, gender, baseline of blood pressure (including SBP and DBP), and coexisting medical conditions (including chronic heart, lung, renal, liver, and cerebrovascular disease, diabetes, and cancer). Detailed information is shown in Supplementary Tables S5–S6.

**Table 2:** Association between antihypertensive use and disease severity of COVID-19 patients with hypertension comorbidity.

					Unadjusted			Adjusted^a^		
Characteristics		Total patients	Mild	Severe	OR	95% CI	*P* value	OR	95% CI	*P* value
All cases, *n* (%)		655	486	169						
	No use	69 (10.5)	44 (9.1)	25 (14.8)	Ref.		Ref.	Ref.		Ref.
	ARB	149 (22.7)	118 (24.3)	31 (18.3)	0.462	0.246–0.869	0.02	0.704	0.489–1.011	0.058
	ACEI	44 (6.7)	35 (7.2)	9 (5.3)	0.453	0.187–1.093	0.094	0.678	0.457–1.01	0.054
	Thiazide	38 (5.8)	28 (5.8)	10 (5.9)	0.629	0.263–1.505	0.39	0.82	0.611–1.102	0.188
	BB	100 (15.3)	77 (15.8)	23 (13.6)	0.526	0.267–1.034	0.082	0.843	0.706–1.006	0.058
	CCB	441 (67.3)	335 (68.9)	106 (62.7)	0.557	0.325–0.953	0.038	0.472	0.257–0.865	0.015
Older than 65 years, *n* (%)		318	209	109						
	No use	31 (9.7)	13 (6.2)	18 (16.5)	Ref.		Ref.	Ref.		Ref.
	ARB	78 (24.5)	56 (26.8)	22 (20.2)	0.284	0.119–0.675	0.005	0.655	0.397–1.081	0.098
	ACEI	19 (6)	15 (7.2)	4 (3.7)	0.193	0.052–0.716	0.018	0.156	0.036–0.67	0.013
	Thiazide	24 (7.5)	17 (8.1)	7 (6.4)	0.297	0.096–0.923	0.055	0.777	0.499–1.211	0.266
	BB	54 (17)	38 (18.2)	16 (14.7)	0.304	0.121–0.765	0.012	0.807	0.64–1.017	0.069
	CCB	214 (67.3)	148 (70.8)	66 (60.6)	0.322	0.149–0.696	0.004	0.287	0.114–0.723	0.008

^a^Fully adjusted model includes the following covariates: age, gender, baseline of blood pressure (including SBP and DBP), and coexisting medical conditions (including chronic heart, lung, renal, liver, and cerebrovascular disease, diabetes, and cancer). Detailed information is shown in Supplementary Tables S7–S8.

### CCB and ACEI are associated with reduced disease severity in elderly COVID-19 patients

We observed statistically significant reductions of disease severity in CCB antihypertensive drug subgroups compared to no drug users after adjustment (adjusted OR = 0.472, 95% CI: 0.257–0.865, *P* = 0.015), especially in elderly patients (adjusted OR = 0.287, 95% CI: 0.114–0.723, *P* = 0.008) (Table 2 and Supplementary Tables S7, S8). The disease severity of ACEI users was statistically significantly reduced in elderly patients (adjusted OR = 0.156, 95% CI: 0.036–0.67, *P* = 0.013) (Table 2 and Supplementary Table S8). Statistically significant reductions in the odds of developing severe disease among ARB users compared to no drug users were observed without adjustment (Table 2). Thus, CCB and ACEI antihypertensive drugs might associate with disease severity in COVID-19 patients.

### ARB associated with the reductions of dyspnea and fatigue in COVID-19 patients

Dyspnea and fatigue are the characteristics of COVID-19. Especially respiratory distress dyspnea, which is defined by a saturation of peripheral oxygen (SPO_2_) <93%, and a respiratory rate >30 times per min. Using the multivariable logistic regression method calculated in cohort C, which recorded some clinically detailed manifestations, we found that ARB users compared to no antihypertensive drug users were associated with reduced dyspnea (adjusted OR = 0.4, 95% CI: 0.236–0.678, *P* = 0.001) (Table 3) and less fatigue (adjusted OR = 0.643, 95% CI: 0.457–0.906, *P* = 0.012) (Table 4). Similar results were also observed in elderly COVID-19 patients (Tables 3 and 4). BB and CCB users were also associated with significantly reduced dyspnea, in COVID-19 patients (Table 3). These data suggest that ARB might improve respiratory syndromes in COVID-19 patients.

**Table 3:** Association between antihypertensive use and dyspnea of COVID-19 patients with hypertension comorbidity.

					Unadjusted			Adjusted^b^		
Characteristics		Total patients	Nonsevere = 0	Severe^a^ = 1	OR	95% CI	*P* value	OR	95% CI	*P* value
Cohort C, *n* (%)		617	525	92						
	No use	59 (9.6)	41 (7.8)	18 (19.6)	Ref.		Ref.	Ref.		Ref.
	ARB	139 (22.5)	127 (24.2)	12 (13)	0.215	0.096–0.484	<0.001	0.4	0.236–0.678	0.001
	ACEI	43 (7)	37 (7)	6 (6.5)	0.369	0.132–1.03	0.061	0.785	0.522–1.179	0.243
	Thiazide	34 (5.5)	30 (5.7)	4 (4.3)	0.304	0.093–0.99	0.046	0.702	0.454–1.086	0.112
	BB	95 (15.4)	80 (15.2)	15 (16.3)	0.427	0.195–0.933	0.043	0.792	0.64–0.981	0.033
	CCB	423 (68.6)	365 (69.5)	58 (63)	0.362	0.195–0.673	0.002	0.283	0.141–0.567	<0.001
Older than 65 years from cohort C, *n* (%)		293	238	55						
	No use	26 (8.9)	13 (5.5)	13 (23.6)	Ref.		Ref.	Ref.		Ref.
	ARB	71 (24.2)	62 (26.1)	9 (16.4)	0.145	0.051–0.41	<0.001	0.415	0.209–0.824	0.012
	ACEI	19 (6.5)	17 (7.1)	2 (3.6)	0.118	0.022–0.615	0.009	0.406	0.142–1.159	0.092
	Thiazide	22 (7.5)	20 (8.4)	2 (3.6)	0.1	0.019–0.518	0.004	0.341	0.091–1.284	0.112
	BB	50 (17.1)	42 (17.6)	8 (14.5)	0.19	0.065–0.56	0.003	0.648	0.47–0.894	0.008
	CCB	201 (68.6)	169 (71)	32 (58.2)	0.189	0.08–0.446	<0.001	0.166	0.058–0.48	0.001

^a^SPO_2_ <93% or respiratory rate 30 times/min.

^b^Fully adjusted model includes the following covariates: age, gender, baseline of blood pressure (including SBP and DBP), and coexisting medical conditions (including chronic heart, lung, renal, liver, and cerebrovascular disease, diabetes, and cancer).

**Table 4:** Association between antihypertensive use and fatigue of COVID-19 patients with hypertension comorbidity.

					Unadjusted			Adjusted^a^		
Characteristics		Total patients	Fatigue = 0	Fatigue = 1	OR	95% CI	*P* value	OR	95% CI	*P* value
Cohort C, *n* (%)		617	340	277						
	No use	59 (9.6)	26 (7.6)	33 (11.9)	Ref.		Ref.	Ref.		Ref.
	ARB	139 (22.5)	88 (25.9)	51 (18.4)	0.457	0.246–0.848	0.018	0.643	0.457–0.906	0.012
	ACEI	43 (7)	22 (6.5)	21 (7.6)	0.752	0.342–1.655	0.549	0.956	0.709–1.289	0.768
	Thiazide	34 (5.5)	24 (7.1)	10 (3.6)	0.328	0.134–0.807	0.018	0.817	0.61–1.095	0.177
	BB	95 (15.4)	50 (14.7)	45 (16.2)	0.709	0.369–1.362	0.324	0.951	0.815–1.11	0.525
	CCB	423 (68.6)	233 (68.5)	190 (68.6)	0.642	0.371–1.112	0.126	0.64	0.36–1.14	0.13
Older than 65 years from cohort C, *n* (%)		293	154	139						
	No use	26 (8.9)	10 (6.5)	16 (11.5)	Ref.		Ref.	Ref.		Ref.
	ARB	71 (24.2)	44 (28.6)	27 (19.4)	0.384	0.152–0.966	0.064	0.518	0.278–0.965	0.038
	ACEI	19 (6.5)	9 (5.8)	10 (7.2)	0.694	0.210–2.301	0.761	0.671	0.374–1.204	0.181
	Thiazide	22 (7.5)	15 (9.7)	7 (5)	0.292	0.088–0.964	0.049	0.637	0.382–1.06	0.083
	BB	50 (17.1)	23 (14.9)	27 (19.4)	0.734	0.279–1.928	0.628	0.887	0.678–1.162	0.385
	CCB	201 (68.6)	106 (68.8)	95 (68.3)	0.56	0.242–1.294	0.212	0.475	0.186–1.209	0.118

^a^Fully adjusted model includes the following covariates: age, gender, baseline of blood pressure (including SBP and DBP), and coexisting medical conditions (including chronic heart, lung, renal, liver, and cerebrovascular disease, diabetes, and cancer).

### ARB associated with the improved clinical indices of infection, inflammation, and thrombus in elderly COVID-19 patients

**Fig. 2:**
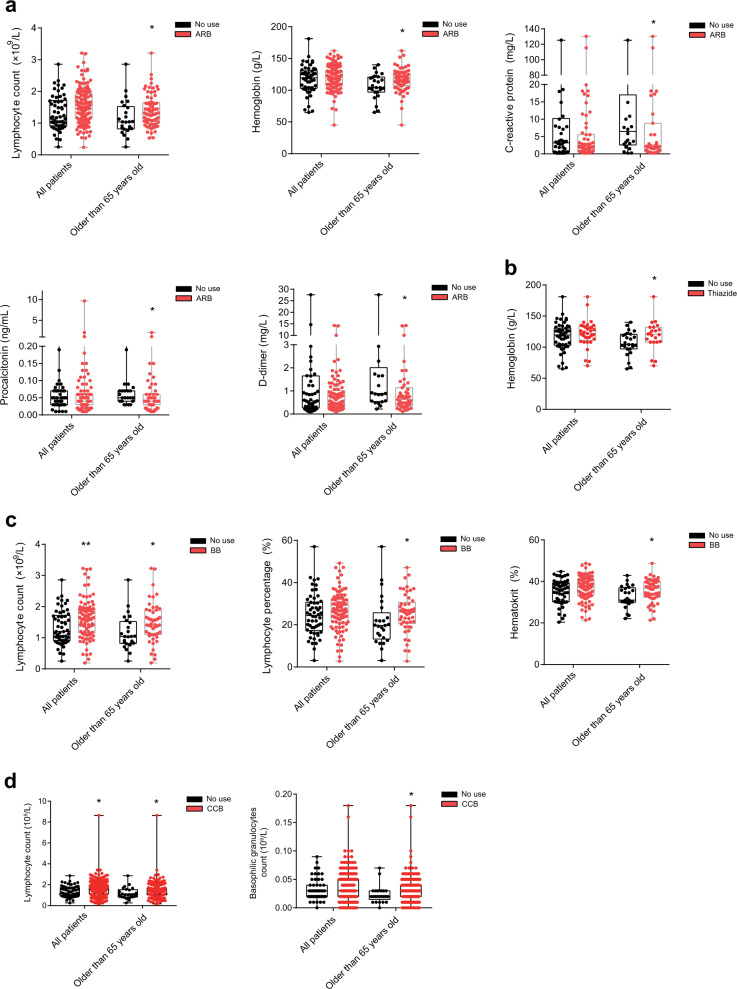
Clinical indices between the use of antihypertensive agents and no use among total cohorts and a subgroup of patients older than 65 years with hypertension comorbidity. **a** Lymphocyte counts, hemoglobin, C-reactive protein, procalcitonin, and D-dimer expression levels of COVID-19 patients between ARB users and no-use antihypertensive agents. **b** Hemoglobin expression levels of COVID-19 patients between used thiazide diuretics and no-use antihypertensive agents. **c** Lymphocyte counts, percentages, and hematocrit expression levels of COVID-19 patients between used BB and no-use antihypertensive agents. **d** Lymphocyte and basophilic granulocyte count expression levels of COVID-19 patients between CCB users and no-use antihypertensive agents, among the total cohort and older than 65-year cohort with hypertension comorbidity. Boxplots with all points are shown in the graph. *P value < 0.05, **P value < 0.01, Mann–Whitney *U* test. Detailed available numbers of each laboratory result group are shown in Supplementary Table S5.

Using the Mann–Whitney *U* test, we discovered that clinical indices related to infection and inflammation, including CRP, lymphocyte count, and procalcitonin, were significantly improved to normal levels in elderly COVID-19 patients who took ARB prior to hospitalization compared to non-antihypertensive drug users (Fig. 2a and Supplementary Table S9). Blood clots have been reported in COVID-19 patients^30,31^. D dimer, a biomarker of blood clots, was significantly reduced in elderly ARB users compared to non-antihypertensive drug users (Fig. 2a). The level of hemoglobin, which carries oxygen from the lungs to the rest of the body, was also significantly increased, returning to normal levels in elderly ARB users (Fig. 2a). COVID-19 elderly patients who took Thiazide, BB, or CCB prior to hospitalization were also associated with these improved clinical indices, except for the blood-clot biomarker (Fig. 2b–d). Taken together, these data indicate that COVID-19 elderly patients with hypertensive comorbidity who took ARB prior to hospitalizations might have reduced clinical syndromes compared to those who took no antihypertensive drugs.

## Discussion

In this retrospective study, we found an association between the use of ARBs and reduced mortality rate in COVID-19 patients with comorbid hypertension (vs those taking no antihypertensives) (Table 1). We also noticed the decreased disease severity in elderly COVID-19 patients taking CCB and ACEI antihypertensive drugs (Table 2).

There are 18 reports studying the association of ARBs and ACEI drugs with COVID-19^26,29,32–47^. Eleven out of the 18 papers reported the analysis of fatal outcome. The results are somewhat controversial due to the different methodologies used and the nature of cohorts. Eight of the eleven papers did not separate ARB and ACEI users, and combined them as one study group. Although both ARBs and ACEI are blockers of RAAS, they target different genes. A more rigorous analysis would be to calculate the COVID-19 association of ARBs and ACEI separately. The other three articles included separated ARBs users’ group^32,42,47^. One of the three reports published in *Hypertension* demonstrating similar conclusion as our report here. However, this study reported in a short communication with only 1 figure without detailed methodology^47^. Two reports published in *JAMA* and *JAMA Cardiology* and one retracted *NEJM* paper show that ARBs were not statistically significantly associated with fatal outcome of COVID-19^32,42,48^. One of the major differences is that their control group was quite different from our controls. The *JAMA* article used CCB users as control group^42^. The other articles (published in *JAMA* *Cardiology* and the retracted *NEJM* article) used all hypertension patients excluding the examined drug users (ARBs nonusers) as their control group^32,48^. Notably, their control group of ARB users included ACEI users. ACEI, as RAAS system drugs, may have similar effects to ARBs. We used their method (i.e., all hypertensive patients excluding ARBs, and including ACEIs in the control group) on our data: the adjusted results showed that ARBs were not statistically significantly associated with fatal outcome of COVID-19. However, ARB drugs statistically significantly improved fatal outcomes of COVID-19 patients when ACEI drug users were removed from the control group (Supplementary Table S10). Thus, this might partially contribute to the different conclusion of this paper that ARBs/ACEI drugs did not statistically significantly reduce the fatal outcomes of COVID-19 patients.

In our study, ARBs and ACEI exhibited different effects associated with the mortality and morbidity of COVID-19 patients, albeit both are RAAS blockers. Previous literature reported that ARBs could effectively block AT1R, whose stimulation is involved in multiorgan injuries^28,49,50^, suggesting that ARBs might be more effective in treating MODS. Besides antihypertensive effects, ARBs could directly reduce lung edema, epithelial and endothelial cell injury, and pro-inflammatory cytokines and chemokines, decrease apoptosis and fibrosis, protect mitochondrial functions, maintain insulin and lipid metabolism, and normalize the coagulation cascade^16,51,52^. These reports may provide mechanisms that ARBs were associated with significantly improved clinical characteristics of COVID-19 patients in this study. Our unpublished data demonstrated that ARBs, especially losartan, enhanced the survival rate more than ACEI in an avian influenza A H5N1 mouse model^16^. Further studies are necessary to elucidate the mechanisms involved in both ACEI and ARBs.

Although a wide variety of conditions, including SARS-CoV, sepsis, acid aspiration, bacteremia, and avian influenza (H5N1 and H7N9) infections, predispose patients to ARDS, a common mechanism seems to be an imbalance of RAAS^9,11–13,23^. Our previous studies in mice suggested that ACE, angiotensin II, and the type 1a angiotensin II receptor participate in disease pathogenesis, whereas ACE2 and the type 2a angiotensin II receptor could protect the mice from severe acute lung injury^9,12^. Blood levels of angiotensin II have been reported to be elevated in patients with COVID-19 (https://www.who.int/news-room/detail/30-01-2020-statement-on-the-second-meeting-of-the-international-health-regulations-(2005)-emergency-committee-regarding-the-outbreak-of-novel-coronavirus-(2019-ncov)) and influenza^9,11^. Interventions that restore the balance of RAAS, for example, ARB antihypertensives, thus could be beneficial. A trial of losartan, a member of ARBs, as treatment for COVID-19 is currently ongoing at the University of Minnesota (NCT043111777 and NCT04312009) (https://clinicaltrials.gov/ct2/show/NCT04311177?cond=COVID-19&lead=University+of+Minnesota&cntry=US&draw=2&rank=2; https://clinicaltrials.gov/ct2/show/NCT04312009?cond=COVID-19&lead=University+of+Minnesota&cntry=US&draw=2&rank=1).

We noted the limitations of this study. The patients’ numbers who took ACEI and thiazide were too small and may induce statistical bias. The outcomes of hypertensive and nonhypertensive COVID-19 patients were not compared. However, the uniqueness of our study is to statistically adjust all combined factors of age, gender, baseline of blood pressure (including SBP and DBP), and coexisting medical conditions (including chronic heart, lung, renal, liver, and cerebrovascular disease, diabetes, and cancer), although BB has no statistical significance before the adjustment.

In summary, the results from this statistical human study link RAAS blockers with COVID-19 patients, and indicate that the use of ARBs in hypertensive patients is associated with decreased mortality of COVID-19 patients. Therefore, hypertensive patients should continue to take ARBs and ACEIs in the current COVID-19 pandemic. Our study also indicates that antihypertensive drugs ARB, ACEI, CCB, and BB might also be potentially economic and effective remedies for COVID-19 patients, especially elderly patients, calling for clinical trials to test this directly.

## Materials and methods

### Study design and participants

In this retrospective study, we identified all the adult patients (≥18 years of age) with laboratory-confirmed COVID-19 pneumonia in the Renmin Hospital of Wuhan University (Wuhan, China) from January 12 to February 9, 2020, the Fifth Medical Center of Chinese PLA General Hospital (Beijing, China) from December 27, 2019 to March 4, 2020, and the Leishenshan Hospital (Wuhan, China) from February 8 to April 14, 2020.

The diagnosis was based on the New Coronavirus Pneumonia Prevention and Control Program published by the National Health Commission of China (2020, http://www.nhc.gov.cn/yzygj/s7653p/202003/46c9294a7dfe4cef80dc7f5912eb1989/files/ce3e6945832a438eaae415350a8ce964.pdf) and WHO interim guidance^53^. Only patients with comorbid hypertension, as diagnosed using the criteria of National Guidelines for Hypertension Management in China^54^ and 2018 ESC/ESH Guidelines for management of arterial hypertension^55^, were included in the final analysis. This study was approved by the Ethics Committees at the Institute of Basic Medical Sciences, Chinese Academy of Medical Sciences (002-2020), and Zhongnan Hospital Ethics Committee (2020067). Verbal informed consent was obtained from patients or their family members if available, and the requirement for written informed consent was waived by the Ethics Committees.

### Data collection

Demographical, epidemiological, clinical, disease severity, and outcome data were extracted from medical records by two trained physicians according to the unified data collection criteria. The classes of antihypertensive medication include ARB, ACE inhibitors, thiazide diuretics, beta-blockers, and calcium-channel blockers, before admission was summarized. Patients diagnosed with hypertension but not taking any antihypertensive drugs were also collected and compared as a control group. Routine blood tests of critically ill patients contained complete blood cell count, serum biochemical examinations (including renal and liver function), coagulation profile, D dimer, interleukin-6 (IL-6), and C-reactive protein (CRP). Disease severity was classified as severe or mild according to the guidelines of 2019-nCoV infection from the National Health Commission of the People’s Republic of China (http://www.nhc.gov.cn/yzygj/s7653p/202003/46c9294a7dfe4cef80dc7f5912eb1989/files/ce3e6945832a438eaae415350a8ce964.pdf). All data were independently checked by more than one physician.

### Definitions

The date of onset was defined as the day when the symptom was noticed. The status at admission and worst status from onset to the final clinical endpoint (discharge/death) of COVID-19 are based on the Chinese official management guidelines for COVID-19 (http://www.nhc.gov.cn/yzygj/s7653p/202003/46c9294a7dfe4cef80dc7f5912eb1989/files/ce3e6945832a438eaae415350a8ce964.pdf): severe COVID-19 comprised any one of the following: at resting condition, the respiratory rate was more than 30 times per minute, blood oxygen was less than or equal to 93%, and an obvious progression of the pulmonary infiltrative lesion as shown by chest radiography was more than 50% within 24–48 h. Hypertension is defined as office SBP values >140 mmHg and/or diastolic BP (DBP) values >90 mmHg according to the 2018 ESC/ESH Guidelines for management of arterial hypertension^55^. Fever was defined as the axillary temperature of at least 37.3 °C.

### Statistical analysis

The Mann–Whitney *U* test was used to compare differences in continuous variables between two groups of COVID-19 cases. The chi-squared test was used to compare differences in categorical variables between two groups of COVID-19 cases. Odds ratio (OR) and 95% confidence interval (CI) were calculated for risk evaluation. Adjusted OR was calculated using “Enter” stepwise multivariable logistic regression. Statistical analyses were performed with SPSS 16.0 for Windows (SPSS, Inc.).

## Supplementary information

Supplementary-revised

## Supplementary information

**Supplementary Information** accompanies the paper at (10.1038/s41421-020-00221-6).

## References

[1] Chan JF-W (2020). A familial cluster of pneumonia associated with the 2019 novel coronavirus indicating person-to-person transmission: a study of a family cluster. Lancet.

[2] Huang C (2020). Clinical features of patients infected with 2019 novel coronavirus in Wuhan, China. Lancet.

[3] Zhang N (2020). Recent advances in the detection of respiratory virus infection in humans. J. Med. Virol..

[4] Hui DS (2020). The continuing 2019-nCoV epidemic threat of novel coronaviruses to global health—the latest 2019 novel coronavirus outbreak in Wuhan, China. Int. J. Infect. Dis..

[5] Wang C, Horby PW, Hayden FG, Gao GF (2020). A novel coronavirus outbreak of global health concern. Lancet.

[6] Zhu N (2020). A novel coronavirus from patients with pneumonia in China, 2019. N. Engl. J. Med..

[7] Liu, Y. et al. Elevated levels of plasma cytokines in the severe acute respiratory syndrome coronavirus 2 (SARS-CoV-2) infection reflect viral load and lung injury. *Natl Sci. Rev.* (2020).

[8] Liu Y (2020). Clinical and biochemical indexes from 2019-nCoV infected patients linked to viral loads and lung injury. Sci. China Life Sci..

[9] Kuba K (2005). A crucial role of angiotensin converting enzyme 2 (ACE2) in SARS coronavirus-induced lung injury. Nat. Med..

[10] Xu Z (2020). Pathological findings of COVID-19 associated with acute respiratory distress syndrome. Lancet. Resp. Med..

[11] Huang F (2014). Angiotensin II plasma levels are linked to disease severity and predict fatal outcomes in H7N9-infected patients. Nat. Commun..

[12] Imai Y (2005). Angiotensin-converting enzyme 2 protects from severe acute lung failure. Nature.

[13] Zou Z (2014). Angiotensin-converting enzyme 2 protects from lethal avian influenza A H5N1 infections. Nat. Commun..

[14] Forrester SJ (2018). Angiotensin II signal transduction: an update on mechanisms of physiology and pathophysiology. Physiol. Rev..

[15] Lin YC (2017). Effects of calcium channel blockers comparing to angiotensin-converting enzyme inhibitors and angiotensin receptor blockers in patients with hypertension and chronic kidney disease stage 3 to 5 and dialysis: a systematic review and meta-analysis. PLoS ONE.

[16] Yan YW (2015). Angiotensin II receptor blocker as a novel therapy in acute lung injury induced by avian influenza A H5N1 virus infection in mouse. Sci. China Life Sci..

[17] Sekizawa K, Matsui T, Nakagawa T, Nakayama K, Sasaki H (1998). ACE inhibitors and pneumonia. Lancet.

[18] Arai T, Yasuda Y, Toshima S, Yoshimi N, Kashiki Y (1998). ACE inhibitors and pneumonia in elderly people. Lancet.

[19] Marciniak C (2009). Examination of selected clinical factors and medication use as risk factors for pneumonia during stroke rehabilitation: a case-control study. Am. J. Phys. Med. Rehabil..

[20] Patel AB, Verma A (2020). COVID-19 and angiotensin-converting enzyme inhibitors and angiotensin receptor blockers: what is the evidence?. JAMA.

[21] Muthiah Vaduganathan OV (2020). Renin-angiotensin-aldosterone system inhibitors in patients with Covid-19. N. Engl. J. Med..

[22] Wang HL (2008). SARS coronavirus entry into host cells through a novel clathrin- and caveolae-independent endocytic pathway. Cell Res..

[23] Sun Y (2015). Cationic nanoparticles directly bind angiotensin-converting enzyme 2 and induce acute lung injury in mice. Part Fibre Toxicol..

[24] Mortensen EM (2012). Population-based study of statins, angiotensin II receptor blockers, and angiotensin-converting enzyme inhibitors on pneumonia-related outcomes. Clin. Infect. Dis..

[25] Zhang P (2020). Association of inpatient use of angiotensin converting enzyme inhibitors and angiotensin II receptor blockers with mortality among patients with hypertension hospitalized with COVID-19. Circ. Res..

[26] Reynolds HR (2020). Renin-angiotensin-aldosterone system inhibitors and risk of Covid-19. N. Engl. J. Med..

[27] Juan Menga GX, Juanjuan Z (2020). Renin-angiotensin system inhibitors improve the clinical outcomes of COVID-19 patients with hypertension. Emerg. Microbes Infect..

[28] Sama IE (2020). Circulating plasma concentrations of angiotensin-converting enzyme 2 in men and women with heart failure and effects of renin-angiotensin-aldosterone inhibitors. Eur. Heart J..

[29] Mancia G, Rea F, Ludergnani M, Apolone G, Corrao G (2020). Renin-angiotensin-aldosterone system blockers and the risk of Covid-19. N. Engl. J. Med..

[30] Gao Y (2020). Diagnostic utility of clinical laboratory data determinations for patients with the severe COVID-19. J. Med. Virol..

[31] Han H (2020). Prominent changes in blood coagulation of patients with SARS-CoV-2 infection. Clin. Chem. Lab. Med..

[32] Li J, Wang X, Chen J, Zhang H, Deng A (2020). Association of renin-angiotensin system inhibitors with severity or risk of death in patients with hypertension hospitalized for coronavirus disease 2019 (COVID-19) infection in Wuhan, China. JAMA Cardiol..

[33] Mehta N (2020). Association of use of angiotensin-converting enzyme inhibitors and angiotensin II receptor blockers with testing positive for coronavirus disease 2019 (COVID-19). JAMA Cardiol..

[34] Hu, J. et al. COVID-19 patients with hypertension have more severity condition, and ACEI/ARB treatment have no infulence on the clinical severity and outcome. *J. Infect.*10.1016/j.jinf.2020.05.056 (2020). 10.1016/j.jinf.2020.05.056 PMC7255723 32474032

[35] Jung, S. Y., Choi, J. C., You, S. H. & Kim, W. Y. Association of renin-angiotensin-aldosterone system inhibitors with COVID-19-related outcomes in Korea: a nationwide population-based cohort study. *Clin. Infect. Dis.*10.1093/cid/ciaa624 (2020). 10.1093/cid/ciaa624 PMC7314113 32442285

[36] Montastruc F (2020). Pharmacological characteristics of patients infected with SARS-Cov-2 admitted to intensive care unit in South of France. Therapie.

[37] Tan ND (2020). Associations between angiotensin converting enzyme inhibitors and angiotensin II receptor blocker use, gastrointestinal symptoms, and mortality among patients with COVID-19. Gastroenterology.

[38] Chen Y (2020). Clinical characteristics and outcomes of patients with diabetes and COVID-19 in association with glucose-lowering medication. Diabetes Care.

[39] Zhou X, Zhu J, Xu T (2020). Clinical characteristics of coronavirus disease 2019 (COVID-19) patients with hypertension on renin-angiotensin system inhibitors. Clin. Exp. Hypertens..

[40] Zhang P (2020). Association of inpatient use of angiotensin-converting enzyme inhibitors and angiotensin II receptor blockers with mortality among patients with hypertension hospitalized with COVID-19. Circulation Res..

[41] Feng Y (2020). COVID-19 with different severities: a multicenter study of clinical features. Am. J. Resp. Crit. Care Med..

[42] Fosbol EL (2020). Association of angiotensin-converting enzyme inhibitor or angiotensin receptor blocker use with COVID-19 diagnosis and mortality. JAMA.

[43] Lam KW (2020). Continued in-hospital ACE inhibitor and ARB use in hypertensive COVID-19 patients is associated with positive clinical outcomes. J. Infect. Dis..

[44] Meng J (2020). Renin-angiotensin system inhibitors improve the clinical outcomes of COVID-19 patients with hypertension. Emerg. Microbes Infect..

[45] Oussalah, A. et al. Long-term ACE inhibitor/ARB use is associated with severe renal dysfunction and acute kidney injury in patients with severe COVID-19: results from a referral center cohort in the North East of France. *Clin. infect. Dis.*10.1093/cid/ciaa677 (2020). 10.1093/cid/ciaa677 PMC7454376 32623470

[46] Peng YD (2020). Clinical characteristics and outcomes of 112 cardiovascular disease patients infected by 2019-nCoV. Zhonghua Xin Xue Guan Bing. Za Zhi.

[47] Zhou F (2020). Comparative impacts of ACE (angiotensin-converting enzyme) inhibitors versus angiotensin II receptor blockers on the risk of COVID-19 mortality. Hypertension.

[48] Mehra, M. R., Desai, S. S., Kuy, S., Henry, T. D. & Patel, A. N. Cardiovascular disease, drug therapy, and mortality in Covid-19. *N. Engl. J. Med.* **382**, e102 (2020). Retracted 10.1056/NEJMoa2007621 PMC7206931 32356626

[49] Palmer SC (2015). Comparative efficacy and safety of blood pressure-lowering agents in adults with diabetes and kidney disease: a network meta-analysis. Lancet.

[50] Dhakarwal P, Agrawal V, Kumar A, Goli KM, Agrawal V (2014). Update on role of direct renin inhibitor in diabetic kidney disease. Ren. Fail..

[51] Saavedra JM (2020). Angiotensin receptor blockers and COVID-19. Pharm. Res..

[52] Gurwitz D (2020). Angiotensin receptor blockers as tentative SARS-CoV-2 therapeutics. Drug Dev. Res..

[53] W. H. O. *Laboratory Testing for 2019 Novel Coronavirus (2019-nCoV) in Suspected Human Cases: Interim Guidance* (World Health Organization, 2020).

[54] Bureau of Disease P, C. N., National Center for Cardiovascular D, et al. (2019). [National guideline for hypertension management in China (2019)]. Zhonghua Xin Xue Guan Bing. Za Zhi.

[55] RE SE, Tsioufis C, Aboyans V, Desormais I (2018). 2018 ESC/ESH guidelines for the management of arterial hypertension. Eur. Heart J..

