# Supplementary material for: Antihypertensive drugs are associated with reduced fatal outcomes and improved clinical characteristics in elderly COVID-19 patients
Source: Cell Discov. 2020 Oct 29;6:77. doi: 10.1038/s41421-020-00221-6 (PMC7595708; doi:10.1038/s41421-020-00221-6)
Supplement: Supplementary file 1 — Supplementary-revised [file 41421_2020_221_MOESM1_ESM.pdf]

Supplementary information

Feifei Yan et al.

Supplementary table S1-S10

Supplementary Table S1. Baseline characteristics of COVID-19 patients with hypertension comorbidity

Characteristics		Total (N=655)	Disease severity			Outcome		
			Mild (N=486)	Severe (N=169)	*P* value	Survial (N=619)	Death (N=36)	*P* value
**Age, mean (SE), y**		64.6 (11.8)	62.9 (11.5)	69.6 (11.0)	<0.001	64.2 (11.7)	71.0 (10.9)	<0.001
**Sex, No.(%)**					0.012			0.17
	**Male**	340 (51.9)	238 (49.0)	102 (60.4)		317 (51.2)	23 (63.9)	
	**Female**	315 (48.1)	248 (51.0)	67 (39.6)		302 (48.8)	13 (36.1)	

Supplementary Table S2. Baseline characteristics of COVID-19 patients (older than 65 years old) with hypertension comorbidity

Characteristics		Total (N=318)	Disease severity			Outcome		
			Mild (N=209)	Severe (N=109)	*P* value	Survial (N=292)	Death (N=26)	*P* value
**Age, mean (SE), y**		74.1 (6.5)	73.1 (5.8)	76.1 (7.3)	<0.001	73.9 (6.5)	76.8 (5.9)	0.012
**Sex, No.(%)**					0.001			0.105
	**Male**	158 (49.7)	89 (42.6)	69 (63.3)		141 (48.3)	17 (65.4)	
	**Female**	161 (50.3)	120 (57.4)	40 (36.7)		151 (51.7)	9 (34.6)	

Supplementary Table S3. Clinical characteristics of COVID-19 patients among different antihypertensive use groups

Characteristics	No use (N=69)	ARB (N=149)	ACEI (N=44)	Thiazide (N=38)	BB (N=100)	CCB (N=441)
**Age, mean (SE), y**	66.42 (13.13)	64.77 (11.64)	62.55 (11.46)	66.03 (10.90)	64.71 (11.99)	64.47 (11.74)
***P* value**	ref	0.35	0.111	0.868	0.382	0.208
**Gender, No. (%)**						
**Male**	43 (62.3)	69 (46.3)	27 (61.4)	20 (52.6)	53 (53)	230 (52.2)
**Female**	26 (37.7)	80 (53.7)	17 (38.6)	18 (47.4)	47 (47)	211 (47.8)
***P* value**	ref	0.03	0.999	0.412	0.27	0.121
**Baseline of blood pressure**						
**SBP, mean (SE), mmHg**	137.01 (17.56)	141.78 (18.98)	141 (20.76)	138.18 (17.80)	144.57 (19.67)	140.89 (17.64)
***P* value**	ref	0.081	0.279	0.747	0.012	0.091
**DBP, mean (SE), mmHg**	82.65 (12.3)	84.74 (12.82)	85.84 (12.94)	84.24 (11.02)	86.25 (12.14)	84.54 (12.52)
***P* value**	ref	0.259	0.191	0.51	0.062	0.243
**Co-existing medical conditions**						
**Chronic heart disease, No. (%)**	4 (5.8)	21 (14.1)	6 (13.6)	17 (44.7)	28 (28)	77 (17.5)
***P* value**	ref	0.108	0.184	0.001	0.001	0.012
**Chronic lung disease, No. (%)**	6 (8.7)	5 (3.4)	2 (4.5)	1 (2.6)	3 (3)	10 (2.3)
***P* value**	ref	0.106	0.48	0.417	0.162	0.013
**Chronic renal disease, No. (%)**	5 (7.2)	12 (8.1)	4 (9.1)	4 (10.5)	10 (10)	49 (11.1)
***P* value**	ref	0.99	0.734	0.718	0.594	0.405
**Chronic liver disease, No. (%)**	0 (0)	3 (2)	2 (4.5)	1 (2.6)	4 (4)	14 (3.2)
***P* value**	ref	0.553	0.149	0.355	0.146	0.234
**Cerebrovascular disease, No. (%)**	5 (7.2)	11 (7.4)	3 (6.8)	5 (13.2)	8 (8)	30 (6.8)
***P* value**	ref	0.99	0.99	0.322	0.99	0.801
**Diabetes, No. (%)**	10 (14.5)	44 (29.5)	12 (27.3)	11 (28.9)	22 (22)	97 (22)
***P* value**	ref	0.018	0.143	0.081	0.238	0.203
**Cancer, No. (%)**	3 (4.3)	7 (4.7)	1 (2.3)	1 (2.6)	0 (0)	8 (1.8)
***P* value**	ref	0.99	0.99	0.99	0.066	0.176

SBP: systemic blood pressure; DBP: diastolic blood pressure

Supplementary Table S4. Clinical characteristics of COVID-19 patients (older than 65 years old) among different antihypertensive use groups

Characteristics	No use (N=31)	ARB (N=78)	ACEI (N=19)	Thiazide (N=24)	BB (N=54)	CCB (N=214)
**Age, mean (SE), y**	78.55 (7.37)	73.12 (6.38)	71.84 (6.28)	72.04 (5.45)	73.65 (5.81)	73.95 (6.24)
***P* value**	ref	0.001	0.002	0.001	0.001	0.001
**Gender, No. (%)**						
**Male**	21 (67.7)	31 (39.7)	13 (68.4)	11 (45.8)	23 (42.6)	106 (49.5)
**Female**	10 (32.3)	47 (60.3)	6 (31.6)	13 (54.2)	31 (57.4)	108 (50.5)
***P* value**	ref	0.011	0.99	0.168	0.042	0.082
**Baseline of blood pressure**						
**SBP, mean (SE), mmHg**	132 (16.96)	142.71 (18.8)	144.16 (21.88)	138.04 (21.3)	145.4 (19.95)	142.25 (18.13)
***P* value**	ref	0.007	0.033	0.247	0.002	0.003
**DBP, mean (SE), mmHg**	79.52 (11.09)	83.32 (10.7)	85.32 (14.03)	82.58 (10.26)	84.74 (13.85)	82.42 (11.64)
***P* value**	ref	0.1	0.111	0.298	0.077	0.193
**Co-existing medical conditions**						
**Chronic heart disease, No. (%)**	3 (9.7)	16 (20.5)	5 (26.3)	15 (62.5)	20 (37)	55 (25.7)
***P* value**	ref	0.264	0.232	0.001	0.01	0.068
**Chronic lung disease, No. (%)**	4 (12.9)	3 (3.8)	0 (0)	1 (4.2)	2 (3.7)	6 (2.8)
***P* value**	ref	0.099	0.284	0.373	0.185	0.026
**Chronic renal disease, No. (%)**	1 (3.2)	3 (3.8)	1 (5.3)	2 (8.3)	5 (9.3)	25 (11.7)
***P* value**	ref	0.99	0.99	0.575	0.409	0.217
**Chronic liver disease, No. (%)**	0 (0)	1 (1.3)	1 (5.3)	0 (0)	1 (1.9)	7 (3.3)
***P* value**	ref	0.99	0.38	——	0.99	0.6
**Cerebrovascular disease, No. (%)**	3 (9.7)	10 (12.8)	0 (0)	4 (16.7)	6 (11.1)	21 (9.8)
***P* value**	ref	0.755	0.279	0.686	0.99	0.99
**Diabetes, No. (%)**	5 (16.1)	25 (32.1)	7 (36.8)	6 (25)	14 (25.9)	54 (25.2)
***P* value**	ref	0.103	0.171	0.505	0.419	0.369
**Cancer, No. (%)**	1 (3.2)	4 (5.1)	1 (5.3)	1 (4.2)	0 (0)	6 (2.8)
***P* value**	ref	0.99	0.99	0.99	0.365	0.99

SBP: systemic blood pressure; DBP: diastolic blood pressure

Supplementary Table S5. Multivariable logistics regression model of association between antihypertensive use and outcome in COVID-19 patients with hypertension comorbidity.

Characteristics	Index	B	S.E.	Wald	Sig.	Adjusted OR	95% CI for Adjusted OR	
							Lower	Upper
**ARB**	Age	0.054	0.035	2.337	0.126	1.055	0.985	1.131
	Gender	1.106	0.871	1.611	0.204	3.021	0.548	16.656
	SBP	-0.005	0.024	0.037	0.848	0.995	0.949	1.044
	DBP	0.030	0.036	0.686	0.408	1.030	0.960	1.106
	Chronic heart disease	0.295	1.125	0.069	0.793	1.343	0.148	12.191
	Chronic lung disease	-18.898	10714.194	0.000	0.999	0.000	0.000	——
	Chronic renal disease	1.812	0.947	3.664	0.056	6.122	0.958	39.142
	Chronic liver disease	-17.166	22022.832	0.000	0.999	0.000	0.000	——
	Cerebrovascular disease	0.72	1.083	0.443	0.506	2.057	0.246	17.193
	Diabetes	0.622	0.855	0.529	0.467	1.862	0.349	9.948
	Cancer	-17.294	11560.160	0.000	0.999	0.000	0.000	——
	ARB	-0.865	0.407	4.526	0.033	0.421	0.190	0.934
	Constant	-8.875	4.829	3.377	0.066	0.000	——	——
**ACEI**	Age	0.091	0.049	3.429	0.064	1.096	0.995	1.207
	Gender	1.589	1.33	1.437	0.231	4.900	0.364	65.894
	SBP	-0.013	0.035	0.148	0.700	0.987	0.922	1.056
	DBP	0.077	0.052	2.153	0.142	1.080	0.975	1.196
	Chronic heart disease	1.710	1.616	1.119	0.290	5.527	0.233	131.366
	Chronic lung disease	-20.037	11785.491	0.000	0.999	0.000	0.000	——
	Chronic renal disease	2.601	1.300	4.000	0.045	13.476	1.053	172.390
	Chronic liver disease	-16.325	27324.892	0.000	1.000	0.000	0.000	——
	Cerebrovascular disease	1.78	1.416	1.589	0.208	5.955	0.371	95.461
	Diabetes	-0.598	1.429	0.175	0.675	0.550	0.033	9.049
	Cancer	-16.940	19403.361	0.000	0.999	0.000	0.000	——
	ACEI	-0.698	0.506	1.902	0.168	0.497	0.184	1.34
	Constant	-14.861	7.234	4.220	0.040	0.000	——	——
**Thiazide**	Age	0.024	0.036	0.5	0.499	1.025	0.955	1.100
	Gender	1.345	0.864	2.420	0.120	3.837	0.705	20.888
	SBP	-0.012	0.024	0.241	0.623	0.988	0.943	1.036
	DBP	0.018	0.039	0.218	0.641	1.018	0.944	1.098
	Chronic heart disease	0.332	0.911	0.133	0.715	1.394	0.234	8.315
	Chronic lung disease	-19.165	14827.830	0.000	0.999	0.000	0.000	——
	Chronic renal disease	1.232	1.058	1.357	0.244	3.430	0.431	27.286
	Chronic liver disease	-19.015	40192.970	0.000	1.000	0.000	0.000	——
	Cerebrovascular disease	1.95	0.874	4.952	0.026	6.997	1.261	38.821
	Diabetes	0.323	0.905	0.127	0.721	1.381	0.234	8.139
	Cancer	-17.801	19759.838	0.000	0.999	0.000	0.000	——
	Thiazide	-0.008	0.203	0.002	0.968	0.992	0.666	1.477
	Constant	-5.106	5.022	1.034	0.309	0.006	——	——
**BB**	Age	0.104	0.051	4.2	0.041	1.109	1.004	1.225
	Gender	0.214	1.081	0.039	0.843	1.238	0.149	10.313
	SBP	-0.043	0.035	1.523	0.217	0.958	0.894	1.026
	DBP	0.053	0.039	1.830	0.176	1.054	0.977	1.138
	Chronic heart disease	3.365	1.594	4.456	0.035	28.942	1.272	658.557
	Chronic lung disease	-0.833	1.436	0.337	0.562	0.435	0.026	7.252
	Chronic renal disease	2.534	1.154	4.819	0.028	12.598	1.312	120.967
	Chronic liver disease	-16.396	17741.421	0.000	0.999	0.000	0.000	——
	Cerebrovascular disease	0.43	1.251	0.121	0.728	1.545	0.133	17.935
	Diabetes	0.394	1.015	0.151	0.698	1.483	0.203	10.846
	Cancer	-16.035	22498.210	0.000	0.999	0.000	0.000	——
	BB	-0.700	0.314	4.968	0.026	0.496	0.268	0.919
	Constant	-9.253	5.938	2.428	0.119	0.000	——	——
**CCB**	Age	0.074	0.022	11.251	0.001	1.077	1.031	1.125
	Gender	0.88	0.470	3.476	0.062	2.401	0.956	6.029
	SBP	0.026	0.013	3.907	0.048	1.026	1.000	1.053
	DBP	0.006	0.020	0.101	0.750	1.006	0.967	1.047
	Chronic heart disease	0.090	0.555	0.026	0.871	1.094	0.369	3.248
	Chronic lung disease	-19.196	9258.127	0.000	0.998	0.000	0.000	——
	Chronic renal disease	1.166	0.532	4.796	0.029	3.209	1.130	9.109
	Chronic liver disease	0.331	1.149	0.083	0.774	1.392	0.146	13.235
	Cerebrovascular disease	1.16	0.557	4.339	0.037	3.189	1.071	9.497
	Diabetes	-0.242	0.544	0.198	0.657	0.785	0.270	2.282
	Cancer	-17.752	11529.166	0.000	0.999	0.000	0.000	——
	CCB	-1.08	0.534	4.084	0.043	0.340	0.119	0.968
	Constant	-12.184	2.767	19.384	0	0.000	——	——

Supplementary Table S6. Multivariable logistics regression model of association between antihypertensive use and outcome in COVID-19 patients (older than 65 years old) with hypertension comorbidity.

Characteristics	Index	B	S.E.	Wald	Sig.	Adjusted OR	95% CI for Adjusted OR	
							Lower	Upper
**ARB**	Age	-0.060	0.076	0.627	0.428	0.942	0.811	1.093
	Gender	1.606	1.201	1.788	0.181	4.982	0.473	52.440
	SBP	0.023	0.034	0.453	0.501	1.023	0.957	1.093
	DBP	-0.037	0.054	0.465	0.495	0.964	0.867	1.071
	Chronic heart disease	-0.006	1.404	0.000	0.996	0.994	0.063	15.559
	Chronic lung disease	-19.297	13100.395	0.000	0.999	0.000	0.000	——
	Chronic renal disease	-17.539	20851.397	0.000	0.999	0.000	0.000	——
	Chronic liver disease	2.279	45279.748	0.000	1.000	9.762	0.000	——
	Cerebrovascular disease	1.08	1.302	0.689	0.407	2.945	0.230	37.763
	Diabetes	2.061	1.192	2.991	0.084	7.851	0.760	81.127
	Cancer	-18.431	15186.303	0.000	0.999	0.000	0.000	——
	ARB	-1.600	0.667	5.764	0.016	0.202	0.055	0.745
	Constant	1.604	7.911	0.041	0.839	4.974	——	——
**ACEI**	Age	0.023	0.103	0.048	0.827	1.023	0.835	1.253
	Gender	-0.581	1.60	0.131	0.717	0.560	0.024	12.956
	SBP	0.061	0.074	0.662	0.416	1.062	0.918	1.230
	DBP	-0.035	0.092	0.142	0.706	0.966	0.807	1.156
	Chronic heart disease	31.915	8967.364	0.000	0.997	72556545681560.200	0.000	——
	Chronic lung disease	-53.941	17823.652	0.000	0.998	0.000	0.000	——
	Chronic renal disease	-19.802	20373.119	0.000	0.999	0.000	0.000	——
	Chronic liver disease	33.408	41763.970	0.000	0.999	322704658099335.000	0.000	——
	Cerebrovascular disease	-16.97	6254.211	0.000	0.998	0.000	0.000	——
	Diabetes	2.832	2.648	1.143	0.285	16.978	0.095	3048.976
	Cancer	-19.608	24089.012	0.000	0.999	0.000	0.000	——
	ACEI	-17.489	3782.333	0.000	0.996	0.000	0.000	——
	Constant	-8.385	12.744	0.433	0.511	0.000	——	——
**Thiazide**	Age	-0.039	0.100	0.154	0.694	0.962	0.791	1.169
	Gender	1.464	1.290	1.287	0.26	4.323	0.345	54.224
	SBP	0.017	0.035	0.233	0.629	1.017	0.949	1.090
	DBP	-0.021	0.069	0.090	0.765	0.980	0.856	1.121
	Chronic heart disease	19.077	9200.856	0.000	0.998	192819396.075	0.000	——
	Chronic lung disease	-37.346	17235.965	0.000	0.998	0.000	0.000	——
	Chronic renal disease	-19.335	21123.041	0.000	0.999	0.000	0.000	——
	Chronic liver disease	——	——	——	——	——	——	——
	Cerebrovascular disease	2.291	1.378	2.762	0.097	9.885	0.663	147.346
	Diabetes	1.56	1.659	0.882	0.348	4.753	0.184	122.872
	Cancer	-17.799	25347.042	0.000	0.999	0.000	0.000	——
	Thiazide	-4.985	2300.214	0.000	0.998	0.007	0.000	——
	Constant	-0.678	10.967	0.004	0.951	0.51	——	——
**BB**	Age	0.027	0.081	0.1	0.735	1.028	0.877	1.204
	Gender	0.315	1.072	0.086	0.769	1.370	0.167	11.208
	SBP	-0.036	0.038	0.923	0.337	0.965	0.896	1.038
	DBP	0.033	0.042	0.635	0.425	1.034	0.953	1.121
	Chronic heart disease	2.939	1.830	2.581	0.108	18.903	0.524	682.227
	Chronic lung disease	-0.684	1.592	0.185	0.667	0.504	0.022	11.414
	Chronic renal disease	-16.943	15067.845	0.000	0.999	0.000	0.000	——
	Chronic liver disease	-18.630	40192.970	0.000	1.000	0.000	0.000	——
	Cerebrovascular disease	0.81	1.228	0.434	0.510	2.246	0.203	24.910
	Diabetes	0.861	1.144	0.567	0.451	2.366	0.251	22.260
	Cancer	-18.032	40192.970	0.000	1.000	0.000	0.000	——
	BB	-0.632	0.316	3.996	0.046	0.531	0.286	0.988
	Constant	-2.333	8.396	0.077	0.781	0.097	——	——
**CCB**	Age	0.082	0.041	4.049	0.044	1.085	1.002	1.175
	Gender	1.12	0.564	3.937	0.047	3.061	1.014	9.243
	SBP	0.042	0.016	6.909	0.009	1.043	1.011	1.076
	DBP	-0.003	0.025	0.011	0.918	0.997	0.950	1.047
	Chronic heart disease	0.006	0.626	0.000	0.993	1.006	0.295	3.429
	Chronic lung disease	-19.695	11962.044	0.000	0.999	0.000	0.000	——
	Chronic renal disease	0.337	0.747	0.203	0.652	1.400	0.324	6.057
	Chronic liver disease	1.229	1.202	1.045	0.307	3.417	0.324	36.027
	Cerebrovascular disease	0.67	0.688	0.942	0.332	1.950	0.506	7.511
	Diabetes	0.105	0.613	0.029	0.864	1.110	0.334	3.695
	Cancer	-17.791	14240.980	0.000	0.999	0.000	0.000	——
	CCB	-1.52	0.645	5.519	0.019	0.220	0.062	0.778
	Constant	-13.960	4.408	10.030	0	0.000	——	——

Supplementary Table S7. Multivariable logistics regression model of association between antihypertensive use and disease severity in COVID-19 patients with hypertension comorbidity.

Characteristics	Index	B	S.E.	Wald	Sig.	Adjusted OR	95% CI for Adjusted OR	
							Lower	Upper
**ARB**	Age	0.070	0.017	17.243	0.000	1.073	1.038	1.109
	Gender	0.689	0.363	3.589	0.058	1.991	0.977	4.060
	SBP	-0.002	0.011	0.037	0.848	0.998	0.976	1.020
	DBP	-0.005	0.017	0.082	0.774	0.995	0.962	1.029
	Chronic heart disease	1.132	0.505	5.018	0.025	3.102	1.152	8.352
	Chronic lung disease	-0.665	0.782	0.724	0.395	0.514	0.111	2.380
	Chronic renal disease	0.778	0.626	1.547	0.214	2.178	0.639	7.421
	Chronic liver disease	-19.521	21750.556	0.000	0.999	0.000	0.000	——
	Cerebrovascular disease	-0.16	0.633	0.066	0.797	0.850	0.246	2.937
	Diabetes	-0.414	0.441	0.881	0.348	0.661	0.278	1.569
	Cancer	1.151	0.762	2.285	0.131	3.162	0.711	14.073
	ARB	-0.352	0.185	3.607	0.058	0.704	0.489	1.011
	Constant	-5.150	2.074	6.163	0.013	0.006	——	——
**ACEI**	Age	0.089	0.025	12.498	0.000	1.093	1.040	1.148
	Gender	1.125	0.56	4.002	0.045	3.080	1.023	9.273
	SBP	-0.004	0.016	0.049	0.824	0.996	0.965	1.029
	DBP	0.039	0.026	2.224	0.136	1.040	0.988	1.095
	Chronic heart disease	-0.024	0.926	0.001	0.979	0.976	0.159	5.996
	Chronic lung disease	-1.405	1.142	1.515	0.218	0.245	0.026	2.299
	Chronic renal disease	0.158	0.946	0.028	0.867	1.172	0.184	7.479
	Chronic liver disease	23.221	27448.825	0.000	0.999	12153430777.644	0.000	——
	Cerebrovascular disease	0.39	0.945	0.167	0.682	1.472	0.231	9.376
	Diabetes	0.336	0.738	0.208	0.649	1.400	0.329	5.950
	Cancer	1.585	1.107	2.052	0.152	4.880	0.558	42.700
	ACEI	-0.388	0.202	3.708	0.054	0.678	0.457	1.01
	Constant	-10.148	3.361	9.114	0.003	0.000	——	——
**Thiazide**	Age	0.081	0.025	10.381	0.001	1.084	1.032	1.139
	Gender	0.794	0.499	2.532	0.11	2.211	0.832	5.878
	SBP	-0.008	0.015	0.265	0.607	0.992	0.963	1.022
	DBP	0.028	0.025	1.230	0.267	1.028	0.979	1.080
	Chronic heart disease	0.528	0.686	0.592	0.442	1.695	0.442	6.505
	Chronic lung disease	-1.337	1.080	1.534	0.216	0.263	0.032	2.179
	Chronic renal disease	0.326	0.848	0.147	0.701	1.385	0.263	7.300
	Chronic liver disease	-19.521	40192.970	0.000	1.000	0.000	0.000	——
	Cerebrovascular disease	0.47	0.760	0.385	0.535	1.603	0.361	7.115
	Diabetes	0.116	0.667	0.030	0.862	1.123	0.304	4.150
	Cancer	0.516	1.248	0.171	0.679	1.675	0.145	19.351
	Thiazide	-0.198	0.151	1.734	0.188	0.82	0.611	1.102
	Constant	-7.808	3.390	5.305	0.021	0.000	——	——
**BB**	Age	0.077	0.020	14.7	0.000	1.080	1.039	1.124
	Gender	0.389	0.419	0.864	0.353	1.476	0.650	3.351
	SBP	0.001	0.013	0.009	0.925	1.001	0.977	1.026
	DBP	-0.017	0.019	0.791	0.374	0.983	0.946	1.021
	Chronic heart disease	0.392	0.518	0.574	0.449	1.480	0.537	4.084
	Chronic lung disease	-0.983	0.859	1.308	0.253	0.374	0.069	2.016
	Chronic renal disease	1.133	0.642	3.117	0.077	3.106	0.883	10.931
	Chronic liver disease	0.489	1.320	0.137	0.711	1.630	0.123	21.671
	Cerebrovascular disease	0.00	0.695	0.000	0.997	1.003	0.257	3.917
	Diabetes	1.008	0.474	4.516	0.034	2.740	1.082	6.942
	Cancer	1.186	1.371	0.747	0.387	3.273	0.223	48.115
	BB	-0.171	0.090	3.580	0.058	0.843	0.706	1.006
	Constant	-5.055	2.449	4.258	0.039	0.006	——	——
**CCB**	Age	0.045	0.010	18.623	0.000	1.046	1.025	1.068
	Gender	0.68	0.233	8.586	0.003	1.979	1.254	3.123
	SBP	0.009	0.008	1.337	0.248	1.009	0.994	1.024
	DBP	-0.013	0.011	1.245	0.264	0.987	0.965	1.010
	Chronic heart disease	0.621	0.282	4.859	0.028	1.861	1.071	3.234
	Chronic lung disease	-0.639	0.625	1.042	0.307	0.528	0.155	1.799
	Chronic renal disease	1.112	0.333	11.179	0.001	3.041	1.584	5.836
	Chronic liver disease	0.385	0.616	0.392	0.531	1.470	0.440	4.916
	Cerebrovascular disease	0.42	0.397	1.108	0.293	1.518	0.698	3.303
	Diabetes	0.432	0.263	2.701	0.100	1.540	0.920	2.577
	Cancer	-0.504	0.829	0.369	0.544	0.604	0.119	3.069
	CCB	-0.75	0.309	5.901	0.015	0.472	0.257	0.865
	Constant	-4.376	1.269	11.888	0	0.013	——	——

Supplementary Table S8. Multivariable logistics regression model of association between antihypertensive use and disease severity in COVID-19 patients (older than 65 years old) with hypertension comorbidity.

Characteristics	Index	B	S.E.	Wald	Sig.	Adjusted OR	95% CI for Adjusted OR	
							Lower	Upper
**ARB**	Age	0.048	0.034	2.007	0.157	1.050	0.982	1.122
	Gender	1.195	0.470	6.470	0.011	3.302	1.315	8.291
	SBP	-0.005	0.015	0.088	0.767	0.995	0.966	1.026
	DBP	-0.002	0.024	0.007	0.935	0.998	0.952	1.046
	Chronic heart disease	0.763	0.648	1.389	0.239	2.146	0.603	7.638
	Chronic lung disease	-0.704	0.954	0.543	0.461	0.495	0.076	3.212
	Chronic renal disease	-0.053	1.357	0.002	0.969	0.948	0.066	13.544
	Chronic liver disease	-19.605	40192.970	0.000	1.000	0.000	0.000	——
	Cerebrovascular disease	0.17	0.700	0.056	0.813	1.180	0.299	4.659
	Diabetes	-0.575	0.555	1.075	0.300	0.563	0.190	1.668
	Cancer	0.586	1.045	0.314	0.575	1.796	0.232	13.916
	ARB	-0.423	0.256	2.736	0.098	0.655	0.397	1.081
	Constant	-3.429	3.522	0.948	0.330	0.032	——	——
**ACEI**	Age	0.272	0.111	5.997	0.014	1.313	1.056	1.632
	Gender	2.059	1.02	4.105	0.043	7.836	1.069	57.422
	SBP	0.042	0.049	0.744	0.388	1.043	0.948	1.148
	DBP	0.047	0.066	0.494	0.482	1.048	0.920	1.193
	Chronic heart disease	-2.305	2.570	0.805	0.370	0.100	0.001	15.360
	Chronic lung disease	-3.579	2.023	3.130	0.077	0.028	0.001	1.471
	Chronic renal disease	-20.474	24997.757	0.000	0.999	0.000	0.000	——
	Chronic liver disease	26.984	40192.970	0.000	0.999	523601147466.403	0.000	——
	Cerebrovascular disease	-3.81	2.073	3.371	0.066	0.022	0.000	1.293
	Diabetes	5.580	2.663	4.390	0.036	265.004	1.434	48974.037
	Cancer	1.904	1.806	1.112	0.292	6.711	0.195	231.101
	ACEI	-1.856	0.744	6.218	0.013	0.156	0.036	0.67
	Constant	-30.976	12.343	6.298	0.012	0.000	——	——
**Thiazide**	Age	0.110	0.059	3.491	0.062	1.117	0.995	1.254
	Gender	1.180	0.696	2.874	0.09	3.255	0.832	12.736
	SBP	-0.012	0.021	0.344	0.558	0.988	0.948	1.029
	DBP	0.042	0.038	1.264	0.261	1.043	0.969	1.123
	Chronic heart disease	0.486	1.021	0.227	0.634	1.626	0.220	12.039
	Chronic lung disease	-1.534	1.353	1.284	0.257	0.216	0.015	3.062
	Chronic renal disease	0.061	1.402	0.002	0.965	1.063	0.068	16.610
	Chronic liver disease	——	——	——	——	——	——	——
	Cerebrovascular disease	0.527	1.025	0.265	0.607	1.694	0.227	12.623
	Diabetes	0.39	0.964	0.166	0.684	1.481	0.224	9.792
	Cancer	-19.341	28224.440	0.000	0.999	0.000	0.000	——
	Thiazide	-0.252	0.226	1.240	0.266	0.777	0.499	1.211
	Constant	-10.783	6.490	2.760	0.097	0.00	——	——
**BB**	Age	0.080	0.046	3.0	0.085	1.083	0.989	1.187
	Gender	0.690	0.551	1.567	0.211	1.994	0.677	5.873
	SBP	-0.004	0.019	0.034	0.853	0.996	0.960	1.034
	DBP	-0.020	0.026	0.605	0.437	0.980	0.932	1.031
	Chronic heart disease	0.426	0.650	0.429	0.512	1.531	0.428	5.468
	Chronic lung disease	-0.887	1.008	0.776	0.379	0.412	0.057	2.967
	Chronic renal disease	1.238	0.983	1.587	0.208	3.448	0.502	23.669
	Chronic liver disease	-20.588	40192.970	0.000	1.000	0.000	0.000	——
	Cerebrovascular disease	0.18	0.850	0.043	0.836	1.192	0.225	6.302
	Diabetes	0.864	0.621	1.936	0.164	2.373	0.703	8.011
	Cancer	-20.039	40192.970	0.000	1.000	0.000	0.000	——
	BB	-0.215	0.118	3.300	0.069	0.807	0.640	1.017
	Constant	-4.418	4.788	0.851	0.356	0.012	——	——
**CCB**	Age	0.079	0.025	9.635	0.002	1.082	1.029	1.137
	Gender	1.25	0.334	14.008	0.000	3.492	1.814	6.723
	SBP	0.012	0.011	1.388	0.239	1.012	0.992	1.034
	DBP	-0.002	0.016	0.016	0.900	0.998	0.967	1.030
	Chronic heart disease	0.571	0.360	2.524	0.112	1.771	0.875	3.583
	Chronic lung disease	-1.073	0.807	1.771	0.183	0.342	0.070	1.661
	Chronic renal disease	1.424	0.508	7.844	0.005	4.152	1.533	11.243
	Chronic liver disease	0.992	0.820	1.463	0.226	2.697	0.540	13.463
	Cerebrovascular disease	0.04	0.499	0.006	0.937	1.040	0.391	2.767
	Diabetes	0.209	0.367	0.324	0.569	1.233	0.600	2.532
	Cancer	-20.016	14798.922	0.000	0.999	0.000	0.000	——
	CCB	-1.25	0.471	7.008	0.008	0.287	0.114	0.723
	Constant	-8.088	2.612	9.590	0	0.000	——	——

Supplementary Table S9. The number of cases involved in figure 2.

		Total population (N)	Older than 65 years old (N)
Lymphocyte count			
	No use	54	25
	ARB	138	71
Hemoglobin			
	No use	54	25
	ARB	138	71
C-reactive protein			
	No use	47	22
	ARB	132	61
Procalcitonin			
	No use	53	23
	ARB	115	57
D-dimer			
	No use	47	21
	ARB	130	55
Hemoglobin			
	No use	54	25
	Thiazide	33	22
Lymphocyte count			
	No use	54	25
	BB	94	49
Lymphocyte percentage			
	No use	54	25
	BB	94	49
Hematokrit			
	No use	54	25
	BB	94	49
Lymphocyte count			
	No use	54	417
	CCB	25	199
Basophilic granulocytes count			
	No use	54	415
	CCB	25	199

Supplementary Table S10. Association between antihypertensive use and outcome of COVID-19 patients with hypertension comorbidity (Diffenent control group)

	Characteristics	Total patients	Survival	Death	Unadjusted			Adjusted[a]		
					OR	95% CI	p value	OR	95% CI	p value
All cases, n (%)		655	619	36						
	No use/ACEI/Thiazide/BB/CCB	506 (77.3)	473 (76.4)	33 (91.7)		ref	ref		ref	ref
	ARB	149 (22.7)	146 (23.6)	3 (8.3)	0.295	0.089-0.974	0.039	0.531	0.282-1.001	0.051
	No use/Thiazide/BB/CCB	465 (71.0)	433 (70.0)	32 (88.9)		ref	ref		ref	ref
	ARB	149 (22.7)	146 (23.6)	3 (8.3)	0.521	0.277-0.98	0.025	0.535	0.293-0.978	0.043

[a]: Fully adjusted model includes the following covariates: age, gender, baseline of blood pressure (including SBP and DBP) and co-existing medical conditions (including chronic heart disease,chronic lung disease, chronic renal disease, chronic liver disease, cerebrovascular disease, diabetes and cancer).
